# Usability and acceptability of virtual reality for chronic pain management among diverse patients in a safety-net setting: a qualitative analysis

**DOI:** 10.1093/jamiaopen/ooad050

**Published:** 2023-07-11

**Authors:** Marika Dy, Kristan Olazo, Courtney R Lyles, Sarah Lisker, Jessica Weinberg, Christine Lee, Michelle E Tarver, Anindita Saha, Kimberly Kontson, Richardae Araojo, Ellenor Brown, Urmimala Sarkar

**Affiliations:** Division of General Internal Medicine, Department of Medicine, University of California San Francisco, San Francisco, California, USA; Center for Vulnerable Populations, Zuckerberg San Francisco General Hospital, University of California San Francisco, San Francisco, California, USA; Division of General Internal Medicine, Department of Medicine, University of California San Francisco, San Francisco, California, USA; Center for Vulnerable Populations, Zuckerberg San Francisco General Hospital, University of California San Francisco, San Francisco, California, USA; Division of General Internal Medicine, Department of Medicine, University of California San Francisco, San Francisco, California, USA; Center for Vulnerable Populations, Zuckerberg San Francisco General Hospital, University of California San Francisco, San Francisco, California, USA; Department of Epidemiology and Biostatistics, University of California San Francisco, San Francisco, California, USA; Division of General Internal Medicine, Department of Medicine, University of California San Francisco, San Francisco, California, USA; Center for Vulnerable Populations, Zuckerberg San Francisco General Hospital, University of California San Francisco, San Francisco, California, USA; Center for Devices and Radiological Health, U.S. Food and Drug Administration, White Oak, Maryland, USA; Office of Minority Health and Health Equity, U.S. Food and Drug Administration, White Oak, Maryland, USA; Center for Devices and Radiological Health, U.S. Food and Drug Administration, White Oak, Maryland, USA; Center for Devices and Radiological Health, U.S. Food and Drug Administration, White Oak, Maryland, USA; Center for Devices and Radiological Health, U.S. Food and Drug Administration, White Oak, Maryland, USA; Office of Minority Health and Health Equity, U.S. Food and Drug Administration, White Oak, Maryland, USA; Center for Devices and Radiological Health, U.S. Food and Drug Administration, White Oak, Maryland, USA; Division of General Internal Medicine, Department of Medicine, University of California San Francisco, San Francisco, California, USA; Center for Vulnerable Populations, Zuckerberg San Francisco General Hospital, University of California San Francisco, San Francisco, California, USA

**Keywords:** virtual reality, medical informatics, information technology, implementation science, qualitative research

## Abstract

**Objective:**

The aim of this study was to understand the usability and acceptability of virtual reality (VR) among a racially and ethnically diverse group of patients who experience chronic pain.

**Materials and Methods:**

Using the Technology Acceptance Model theory, we conducted semistructured interviews and direct observation of VR use with English-speaking patients who experience chronic pain treated in a public healthcare system (*n* = 15), using a commercially available VR technology platform. Interviews included questions about current pain management strategies, technology use, experiences and opinions with VR, and motivators for future use.

**Results:**

Before the study, none of the 15 participants had heard about or used VR for pain management. Common motivators for VR use included a previous history of substance use and having exhausted many other options to manage their pain and curiosity. Most participants had a positive experience with VR and 47% found that the VR modules distracted them from their pain. When attempting the navigation-based usability tasks, most participants (73%–92%) were able to complete them independently.

**Discussion:**

VR is a usable tool for diverse patients with chronic pain. Our findings suggest that the usability of VR is not a barrier and perhaps a focus on improving the *accessibility* of VR in safety-net settings is needed to reduce disparities in health technology use.

**Conclusions:**

The usability and acceptability of VR are rarely studied in diverse patient populations. We found that participants had a positive experience using VR, showed interest in future use, and would recommend VR to family and friends.

## INTRODUCTION

Virtual reality (VR) has proven to be a safe and effective pain management strategy.^1–4^ In general, patients consider VR to be a positive experience in managing their pain.^5^ However, most studies were conducted in primarily White, well-resourced settings. It is unclear whether VR is a viable pain management strategy among historically marginalized patients, who already face disparities in pain management.^6^

Among patients with chronic pain, Black or African American patients report significantly higher pain severity compared to White patients.^7–12^ Despite this higher burden of pain, Black or African American patients are 22% less likely than White patients to receive pain medication due to physician perceptions, bias, and racism.^13^ Furthermore, uninsured patients, who come from low socioeconomic backgrounds and identify as a minority are more likely to experience delays in care and poorly coordinated care.^14^ These patient groups are also less likely to have access to primary care,^14^ where most chronic pain is managed in the United States.^15^

Medication therapy for chronic pain has significant risks. Nonopioid pain medications can cause serious adverse events,^16^ and opioid misuse has reached epidemic proportions in the United States.^17^^,^^18^ This leaves patients with chronic pain in a vulnerable position if providers fail to prescribe and monitor medications carefully. Policies to enhance opioid safety have had the unintended consequence of leaving some patients with chronic pain untreated, as they now face greater difficulty accessing medication for pain.^19^ Safer, effective alternatives to manage chronic pain are needed, particularly for patients who already face access barriers to adequate care and disparities in opioid overdose deaths.^20^

As patients are interested in using digital tools to manage their health,^21^^,^^22^ and clinicians are interested in VR as an adjunct or replacement pain management treatment,^23^ VR may be a useful option for many patients with chronic pain. However, despite the benefits of portability, and the potential for an effective, safer treatment, VR has yet to be integrated as an alternative for treating chronic pain. A recent study assessing barriers to VR implementation in safety-net settings found that providers faced challenges to VR adoption, including lack of reimbursement, concerns that existing VR content may not be relevant to diverse patients, and integrating VR into current clinical workflows.^23^

Additionally, in the existing literature, there are limited studies that have evaluated the usability of VR among racially, ethnically, and/or socioeconomically diverse patients.^24^ A recent study examined the usability and acceptability of VR for nutrition education among adult-child dyads with low income.^25^ This evidence gap poses a barrier to VR uptake in these populations as well as the integration of VR as a treatment within safety-net settings, which disproportionately care for non-White patients and those who are uninsured or have low income. To evaluate VR’s usability among patient groups who receive care in the safety-net setting and could potentially benefit from this technology, more research must be done. Device designers should assess the quality of a user’s experience as they interact with the product to adapt these tools to be more appropriately and effectively designed for the device users.^26^ To address this gap in research, we aimed to characterize usability barriers and facilitators of VR for chronic pain management.

## MATERIALS AND METHODS

### Study design

We conducted usability testing to examine VR for pain management among patients who receive care within a public healthcare system. We used semistructured, qualitative interviews to collect information about technology use, current pain management approaches, challenges with pain management, and opinions about VR (see Supplementary Appendix S1). We conducted direct observations of participants while they used the VR headset and completed usability tasks. We also asked participants about their pain before and after using the VR headset using the following question, “Please rate your pain by indicating the number that best describes your pain on average in the last 24 hours, with 0 meaning ‘No pain’ and 10 meaning ‘Pain as bad as you can imagine’”^27^—to explore patient-reported pain in an exploratory way within our usability testing sample. We obtained institutional review board approval (# 19-29025) for this study from the University of California, San Francisco (UCSF).

### Setting and participants

This study took place at a publicly funded, urban, safety-net health system. We recruited participants via an existing pain management registry list from April 2021 to September 2021. We queried this registry through electronic health records to identify patients who (1) had established primary care within the network and (2) had been prescribed an opioid for greater than 90 days and seen in the Pain Clinic within the past 6 months. Per health system policy, we contacted participants’ primary care providers to review their patient’s eligibility for our study and ask for permission to contact their patients. Patients were recruited using convenience sampling. Patients were eligible for the study if they were (1) over 18 years of age, (2) English speaking, (3) not currently incarcerated, (4) had the cognitive ability to consent, and (5) had no history of seizure disorder, vertigo, or claustrophobia.

### VR platform

Participants used the RelieVRx system (AppliedVR, Los Angeles, CA), an FDA-authorized digital therapeutic for chronic lower back pain (Supplementary Figure S1). The RelieVRx system is typically used as an 8-week curriculum that incorporates relaxation-response exercises, biopsychosocial pain education, mindfulness exercises, executive functioning games, and diaphragmatic breathing training, ranging from 2 to 16 minutes in length. For our study, only a subset of the available modules was used to assess user experience and usability in 1 session (Supplementary Figure S2). We selected 3 modules that focused on pain distraction, breathing, and mindfulness for a total of 14 minutes of platform use conducted in that 1 research session. Shorter modules were selected based on literature suggesting that 15–30 minutes of continuous VR use is optimal.^28^ In addition, we wanted to ensure participants were able to experience different types of modules. The immersive therapeutic system includes a VR headset and hand-held controller. The system also offers hands-free, gaze-based control. For this study, we had participants use gaze-based control to navigate the VR content. We chose gaze-based control as an interaction method because it allows for faster pointing,^29^^,^^30^ reduces arm fatigue,^31^ and frees the hand for other tasks.^31^^,^^32^

### Analysis

**Figure 1.**
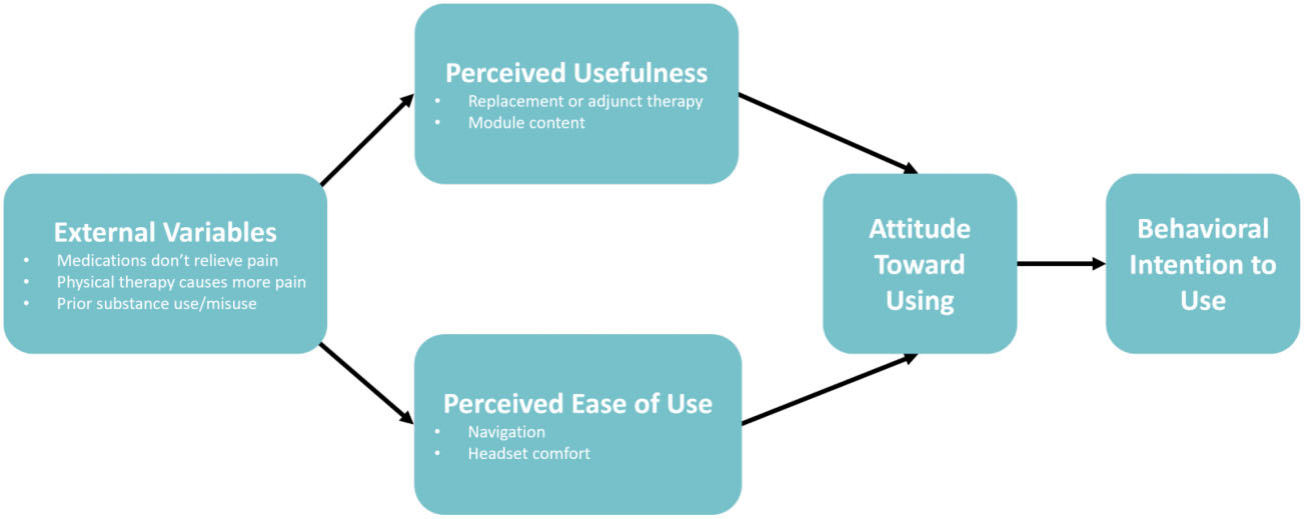
Technology acceptance model.

We used the qualitative software Dedoose (Los Angeles, CA) to conduct our analysis. Two authors (MD and KO) independently read through 4 transcripts to identify themes that emerged from the interviews. We then developed a preliminary codebook based on these themes and applied them to the remaining transcripts. We subsequently met regularly in the larger research team meetings to discuss any new inductive codes that emerged from the transcripts. After we completed coding all transcripts, we systematically reviewed the codes and read the accompanying excerpts in greater detail to identify thematic clusters. We continued recruiting participants until thematic saturation was reached. Cluster themes were then grouped under the Technology Acceptance Model (TAM) (Figure 1). TAM was adapted from 2 psychological theories, the theory of reasoned action and the theory of planned behavior. TAM asserts that a user’s acceptance of technology is influenced directly by 2 main factors: perceived ease of use and perceived usefulness,^33^^,^^34^ alongside external variables that impact perceptions (such as existing treatment/care management practices).

In addition to employing an inductive-deductive approach to understanding the acceptance of VR for pain management, we focused on predetermined tasks to assess for task completion (Table 1). We categorized levels of task completion based on a previous usability study we conducted.^35^ Our usability test focused on participants’ ability to navigate through each session using gaze-based control. All 3 modules required navigating through the same number of tasks: (1) library, (2) specified curriculum (eg, pain distraction), (3) specified module (eg, “Hocus Focus Immersion”), and (4) library. For each session, participants attempted to complete all tasks before needing direct intervention from the interviewer. Although there is not a gold standard, many developers use a cut-off of 70% task completion on the first attempt as a useful metric for usability.^36^

**Table 1.** Definitions for each completion type

Completion type	Definition of each completion type
Successfully completed	Participant immediately navigates to the correct screen and selects the appropriate module after receiving verbal instructions once.
Successfully completed with help	Participant has clarifying questions about the appearance of a menu or page, but not questions about actions that need to be taken. The participant eventually completes the task without direct intervention from the interviewer.
Partial	After providing verbal instructions twice, the participant can navigate to the correct screen on their own.
Unsuccessful	Participant requires interviewer’s direct intervention/guidance after at least two unsuccessful attempts to find the correct module.

## RESULTS

We received a list of 154 prospective English-speaking participants from our query. We then reached out to the primary care providers of 148 prospective participants (6 individuals did not have an assigned PCP). We received a response for 86 patients. Based on our eligibility criteria, 19 individuals were deemed ineligible by their PCP. We received permission to contact 67 prospective participants. During our outreach, 7 were deemed ineligible after screening for history of seizure disorder, vertigo, or claustrophobia. Forty-five either declined to participate or did not answer our calls after 3 attempts. In total, we recruited 15 participants with chronic pain in any part of the body between April 2021 to September 2021. The majority were male (67%), and half the participants identified as Black or African American (Table 2).

**Table 2.** Participant demographics

Characteristics	Overall, *N* = 15, *n* (%)
Age (mean ± SD)	55.80 (9.99)
Sex	
Male	10 (67)
Female	5 (33)
Sex at birth	
Male	10 (67)
Female	5 (33)
Gender	
Male	10 (67)
Female	5 (33)
Nonbinary	0 (0)
Ethnicity	
Hispanic/Latino	2 (13)
Not Hispanic/Latino	13 (87)
Race^a^	
White or Caucasian	3 (21)
Black or African American	7 (50)
Asian	1 (7)
Native Hawaiian/Pacific Islander	1 (7)
American Indian/Alaskan Native	2 (14)
Other	3 (21)
Education^b^	
No high school	0 (0)
Some high school	0 (0)
High school	7 (50)
Some college	3 (21)
College degree	3 (21)
Graduate degree	1 (7)
Comfort filling out medical forms	
Not at all	2 (13)
A little bit	1 (7)
Somewhat	6 (40)
Quite a bit	2 (13)
Extremely	4 (27)

*Abbreviation*: SD: standard deviation.

a Participants could provide multiple responses. *N* = 14 (1 participant did not respond).

b *N* = 14 (1 participant did not provide a response).

Participants with chronic pain perceived the VR intervention as useful and acceptable. Before participating in the study, no participants had heard about or used VR for pain management, but the majority were interested and willing to try it. After using the VR headset, 93% (14/15) of participants said they would recommend VR to their family and friends.

### External variables influencing perceived usefulness and perceived ease of use

Participant experiences with existing pain management strategies were a key factor in the perceived usefulness of VR. In discussing current strategies, 87% (13/15) reported using prescribed or over-the-counter medication to manage their chronic pain. However, 67% (10/15) of unique participants mentioned facing various challenges with their medication: 40% (6/15) reported that their medications fail to relieve their pain, 20% (3/15) reported not wanting to be prescribed opioids due to the previous history of drug addiction, and 13% (2/15) mentioned their provider wanting to change their medication dosage.

*I was on opiates for a while, but opiates don’t do it for me. The best thing that does it for me is ibuprofen, but I can’t take ibuprofen because it messes with my stomach so badly. [PTID75, 47 years old, White male]*

Additionally, 53% (8/15) reported participating in physical therapy or exercise as a current approach to managing their pain, although 27% (4/15) of participants mentioned having a difficult time staying active due to exercise causing more pain. As a result, most participants were open to exploring alternative pain management strategies, citing key motivators such as a previous history of substance use and/or abuse, and having exhausted all other methods to manage their pain.

*If I’m going to my physical therapy and stuff and she does my exercise, then I’m in pain using muscles I haven’t been using in over ten years I can’t stand. I can stand for maybe eight seconds or seven seconds. [PTID82, 62 years old, Black male]*

**Figure 2.**
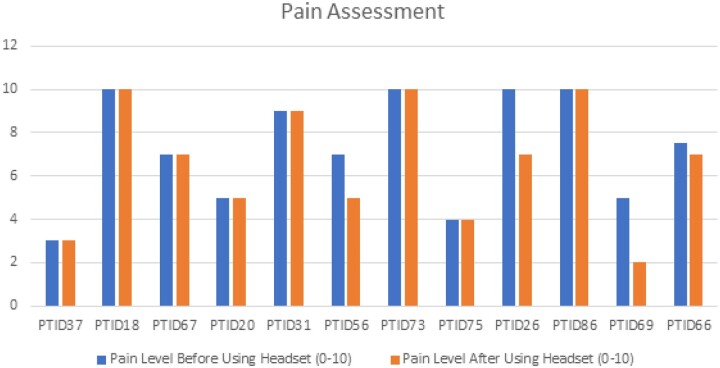
Pain assessment of participants before and after use of virtual reality. Missing data for 3 participants pt88, pt82, and pt83.

In our exploratory analysis, when we asked participants to describe their pain in the last 24 hours, the average rating was 7.3 before using the VR headset, with 3 being the lowest number reported and 10 being the highest number reported (Figure 2). When we asked participants to describe their pain after using the VR headset, 9 participants’ pain levels remained unchanged; 3 participants rated their pain lower after using the VR headset.

### Perceived usefulness: pain distraction

Overall, 47% (7/15) of participants reported that the VR modules distracted them from their pain. Participants commented that the modules helped them focus on something other than their pain. However, of those who reported distraction, 43% (3/7) mentioned that once the VR headset is removed, their pain came back despite having been distracted from it while wearing the headset. Conversely, 57% (4/7) of participants who reported distraction noted that they felt calming after-effects on their pain after using VR.

*Unbelievable. It distracted me. Everything went away. I was in a whole different world. [PTID26, 63 years old, Hispanic/Latino male]*

*…while I was using the system and my mind was focused, I didn’t feel anything. But now that I’m back and I’m not using anything, we go back to reality, my knees said, “Hey, I’m here, buddy. Let’s get going,” you know? [PTID18, 43 years old, Pacific Islander male]*

### Perceived usefulness: adjunct therapy

Although 13% (2/15) of participants viewed VR as a useful and acceptable replacement for their existing pain management strategies, 27% (4/15) could not see it as a replacement.

*I wouldn’t go that far [to replace my pain medication with VR] … the limited amount of use, but I imagine it could. [PTID75, 47 years old, White male]*

Twenty-seven percent (4/15) of participants were unsure if VR could be a replacement strategy, citing reasons such as individual preferences and requiring extended use. Sixty percent (9/15) of participants were more open to using VR as a supplement to their existing pain management strategies.

*I mean, it’s a tool that helps but I don’t think that it could 100% replace the regimen that they got me on… It would just be a perk. Luxury is what it would be. [PTID18, 43 years old, Pacific Islander male]*

### Perceived ease of use: comfort

When we asked participants to provide feedback on how the headset fit and felt on their heads, most participants expressed that the headset was comfortable to wear.

*It’s not too heavy. It’s just right. It’s just the right fit. [PTID31, 61 years old, Black female]*

Two participants commented on the weight of the headset. They thought that the headset was a little heavy, and consequently, they were hyperaware that they were wearing the headset. Participants recommended a lighter and thinner design.

*If they could make something a little thinner or a little lighter too – as you could get into your breathing and your everything, get more relaxed, and if that thing was a little bit lighter, you could completely forget about wearing it. [PTID20, 68 years old, White female]*

### Perceived ease of use: navigation

**Figure 3.**
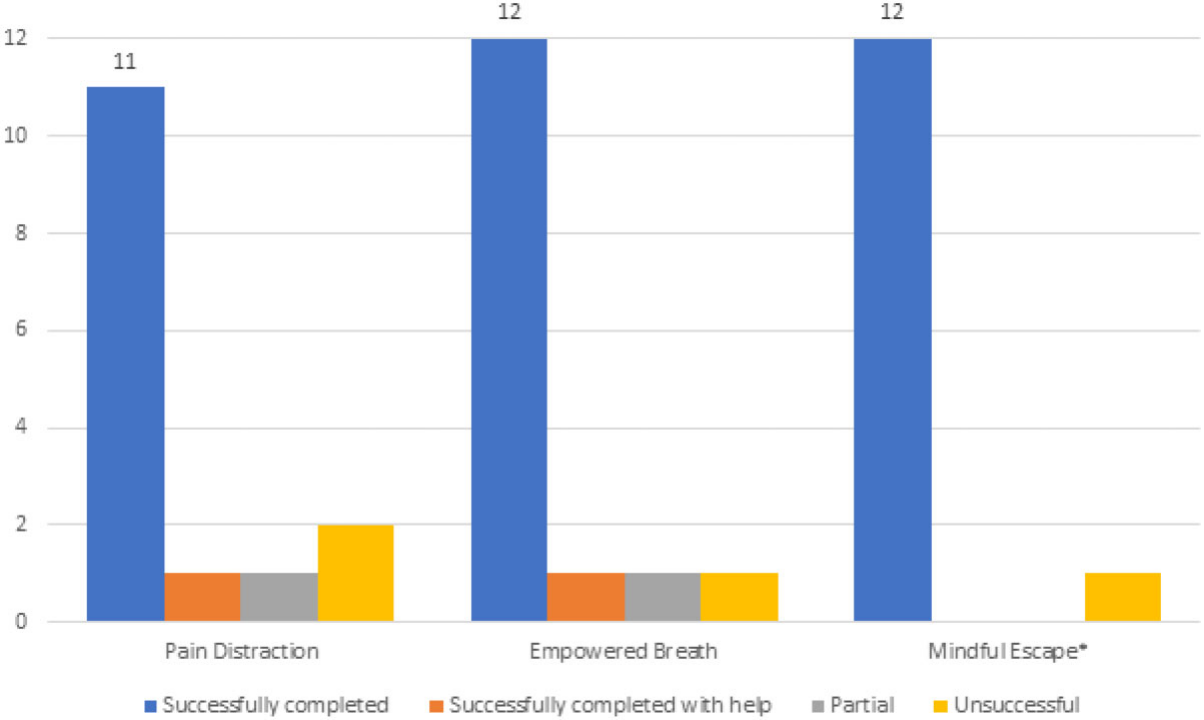
VR usability tasks. *Two participants decided to stop and did not complete the module.

For the pain distraction module, 73% (11/15) of participants were able to complete the navigation-based tasks independently; 13% (2/15) of participants were able to complete the tasks “partially or with assistance,” with clarifying questions or repeated instructions from the interviewer; and 13% (2/15) required the interviewer’s direct intervention to complete the task. For the breathing module, 80% (12/15) of participants were able to complete the tasks independently; 13% (2/15) completed tasks “partially or with assistance”; and 7% (1/15) required the interviewer’s direct intervention to complete the task. For the mindfulness module, 2 participants chose not to complete the module. Of the participants who completed the module, 92% (12/13) completed all tasks independently and 8% (1/13) of participants were unable to complete the tasks without the interviewer’s direct intervention (Figure 3).

*It’s perfect. Just go to the library and select what you need. [PTID69, 58 years old, Hispanic/Latino male]*

*I probably would need help getting started. [PTID56, 60 years old, Black female]*

## DISCUSSION

Patients have demonstrated interest in using digital tools, such as VR, to manage their health.^37–39^ Our findings show that VR may be a useful tool for diverse patients experiencing chronic pain. Future studies should further explore VR use in the safety-net healthcare setting. Most participants were able to complete the navigation-based tasks without any help. In the context of TAM, this suggests that perceived ease of use (ie, usability) of VR was not a barrier for most participants in this study. Instead, and moving beyond ease of use and usefulness, (1) improving awareness and integration of VR in safety-net settings and (2) ensuring that implementation will work in patients’ homes/contexts, is needed to address the widening gap in health technology use.^40–42^ We hope that future researchers employ implementation science research designs and methods, such as a hybrid type 2 implementation trial to assess the effectiveness, feasibility, and implementation of VR for chronic pain in a safety-net healthcare setting,^43^ to better understand VR’s impact for clinical use.

Although more than half of the participants were either unsure or did not see VR replacing their existing pain management strategies, the majority did think it could be a useful tool to supplement their treatment. For example, among almost half of the participants, VR use may be an effective distraction from pain. This finding is similar to that of randomized trials that found VR to be an effective distraction technique in patients with chronic back pain and breast cancer pain.^2^^,^^3^

Similarly, the influence of external variables, including the patient’s history of pain, technology use, and pain management strategies, impacted participants’ perception of how beneficial VR would be for them. Many faced challenges related to their pain management, having exhausted other treatment options, and some even choosing to endure the pain without treatment. As such, participants were curious and willing to try a new approach that could potentially help with their pain. This experience is consistent with a prior study examining acupuncture for chronic pain among a racially/ethnically diverse patient population.^44^ However, some participants were hesitant about the therapeutic effects of VR, due to the “type of pain” they have, suggesting that some participants’ perception of their pain severity may directly impact their attitude toward trying VR. A review of pain beliefs emphasized that patients’ beliefs influence their pain perception and response to treatment.^45^

### Limitations

Our study has some key limitations. First, although we attempted to recruit a diverse sample in our study, half of our participants received a college education, which may have potentially biased our results in terms of participants’ perceived usability of VR. In addition, most participants in our study were male and, therefore, our findings may not be representative of all chronic pain patients. Past studies have shown that women are more likely to experience chronic pain^46^ but are less likely to be prescribed analgesics.^47^ Although we attempted to recruit a balanced sample, we found that many eligible female participants declined to participate; future studies should focus on recruitment strategies to achieve a representative sample. Second, we included only English-speaking patients, excluding a large portion of patients at our study site whose primary language is not English. We included English-speaking participants because the VR content was only available in English. Third, because usability testing involved interviewing as a data collection method, participants may have been hesitant to be completely honest in their feedback due to social desirability bias. Furthermore, our navigation method (ie, using gaze-based control vs hand-held controller) may have impacted the usability of VR for some participants since a gazed-based control may not be as commonly used as a hand-held controller. Our qualitative study reached thematic saturation and identified key themes for the usability of VR, and future studies may focus on the generalizability of these findings within a larger sample. Additionally, the device used in our study is clinically demonstrated to decrease pain for patients with chronic lower back pain. Study participants were not limited to those experiencing chronic lower back pain; hence usability could be affected by the device’s application to a broader population than was intended. We only assessed the usability of 1 user interface. Future studies should evaluate other VR devices for technology acceptance to be more generalizable. We asked participants to rate their pain in the last 24 hours before and after using the headset as opposed to asking participants to rate their pain “right now.” This may have skewed participants’ pain rating after using the headset. Last, our sampling was based on opioid use because our health system has an electronic registry for patients receiving chronic opioid prescriptions, and therefore this group of patients was more reliably identified than the broader group of patients with chronic pain, which can be challenging to accurately identify using the electronic health record.

## CONCLUSION

The objective of our study was to assess the usability and acceptability of VR among patients diverse in race, ethnicity, education, and health literacy. We found that participants had a positive experience using VR, showed interest in future use, and would recommend VR to their family and friends. Future studies should evaluate the use of VR for chronic pain among diverse populations in real-world settings, including engagement, effectiveness, and sustainability of VR for chronic pain treatment.

## DISCLAIMER

This study reflects the views of the authors and should not be construed to represent FDA’s views or policies. The mention of commercial products, their sources, or their use in connection with material reported herein is not to be construed as either an actual or implied endorsement of such products by the Department of Health and Human Services.

## Supplementary Material

ooad050_Supplementary_Data

## Data Availability

The data underlying this article will be shared on reasonable request to the corresponding author.
