# Supplementary material for: Usability and acceptability of virtual reality for chronic pain management among diverse patients in a safety-net setting: a qualitative analysis
Source: JAMIA Open. 2023 Jul 11;6(3):ooad050. doi: 10.1093/jamiaopen/ooad050 (PMC10336187; doi:10.1093/jamiaopen/ooad050)
Supplement: ooad050_Supplementary_Data [file ooad050_supplementary_data.zip › Appendix 2_headset images.docx]

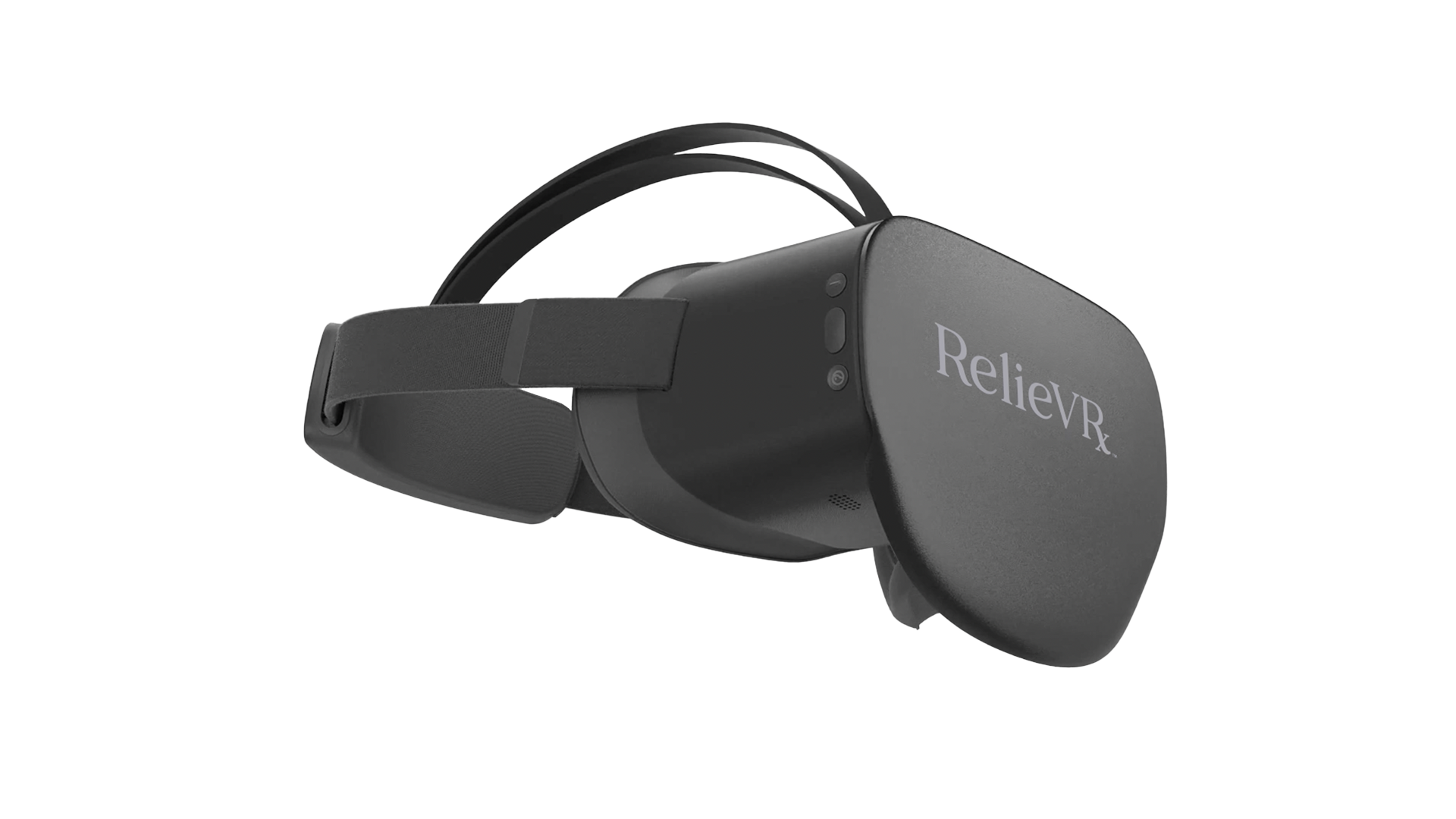


**Figure 1. The RelieVRx™ system headset. Image courtesy of AppliedVR™.**


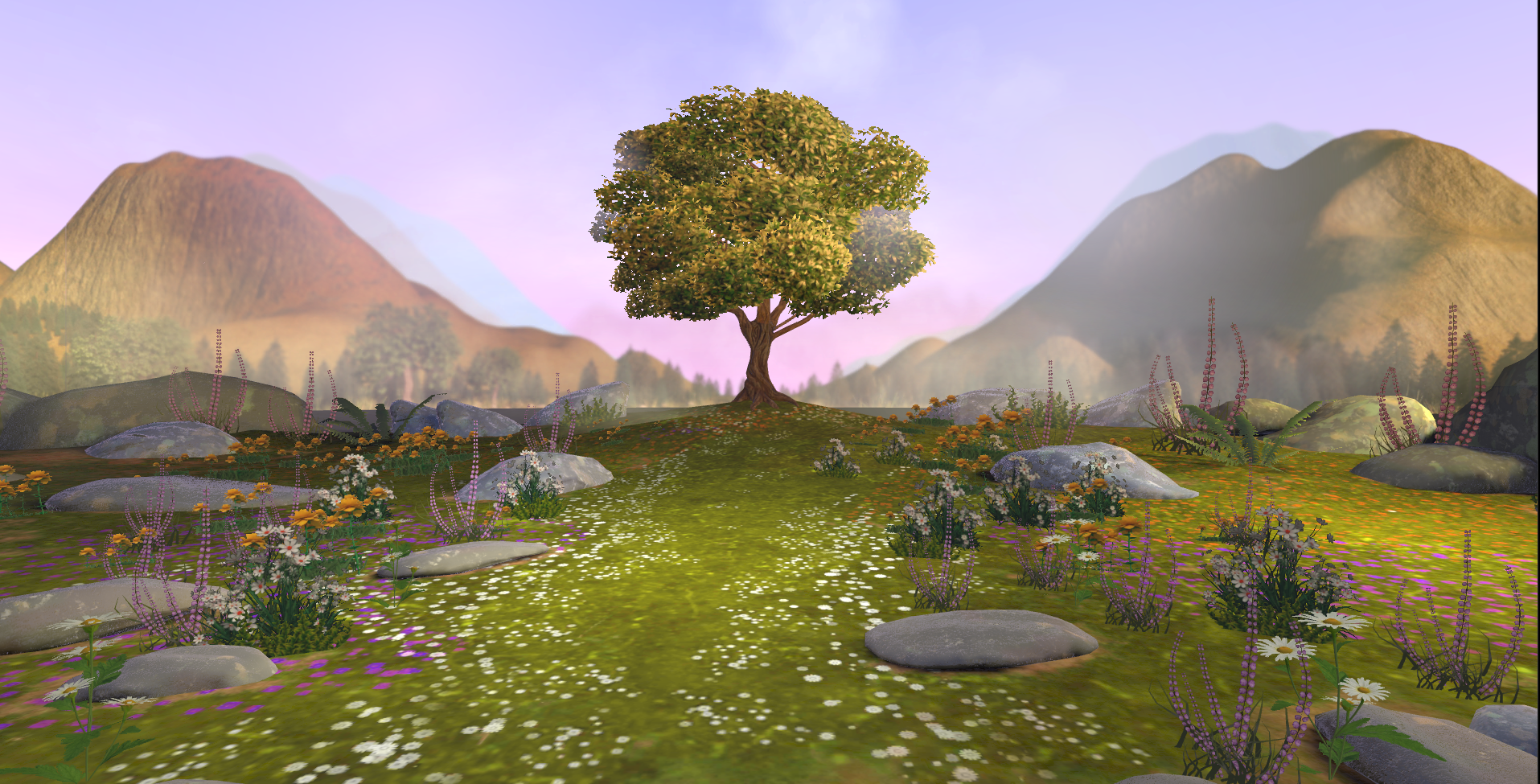


**Figure 2. Example module from the RelieVRx™ system used to assess user experience and usability. Image courtesy of AppliedVR™.**
