# Supplementary material for: Usability and acceptability of virtual reality for chronic pain management among diverse patients in a safety-net setting: a qualitative analysis
Source: JAMIA Open. 2023 Jul 11;6(3):ooad050. doi: 10.1093/jamiaopen/ooad050 (PMC10336187; doi:10.1093/jamiaopen/ooad050)
Supplement: ooad050_Supplementary_Data [file ooad050_supplementary_data.zip › Appendix_Interview and Usability Testing Guide_Patients.docx]

**OMB Control No**: 0910-0497 **Expiration Date**: 10/31/2020

Paperwork Reduction Act Statement: According to the Paperwork Reduction Act of 1995, an agency may not conduct or sponsor, and a person is not required to respond to a collection of information unless it displays a valid OMB control number. The valid OMB control number for this information collection is 0910-0497. The time required to complete this information collection is estimated to average 75 minutes per response, including the time for reviewing instructions, searching existing data sources, gathering and maintaining the data needed, and completing and reviewing the collection of information.

Send comments regarding this burden estimate or any other aspects of this collection of information, including suggestions for reducing burden to [PRAStaff@fda.hhs.gov](mailto:PRAStaff@fda.hhs.gov).

**Qualitative Interview and Usability Testing Guide**

**Patients**

**Study Title**: Usability Testing of Virtual Reality for Opioid-Sparing Pain Management Among Diverse Patients

**Step 1. Consent Study Participant**

- Review consent document with study participant and obtain signature

**Step 2. Provide Study Participant with Incentive Payment**

- Have participant complete/sign acknowledgement of payment
- Give participant payment

**Step 3. Background Demographic/Technology Questions:** Let participant know you will start with some background questions.

1. What is your age?
2. What is your sex?
   1. Male
   2. Female
   3. Unknown
3. What is your sex at birth?
   1. Male
   2. Female
   3. Prefer not to answer
   4. Don’t know
4. What is your gender?
   1. Male
   2. Female
   3. Non-binary/ third gender
   4. Prefer to self-describe
5. What best describes your ethnicity?
   1. Hispanic or Latino
   2. Not Hispanic or Latino
6. Race. Which of the following best describes your race? Mark one or more
   1. White
   2. Black or African American
   3. Asian
   4. Native Hawaiian or Other Pacific Islander
   5. American Indian or Alaska Native
7. How comfortable are you filling out medical forms by yourself?
   1. Not at all
   2. A little bit
   3. Somewhat
   4. Quite a bit
   5. Extremely
8. What is your highest level of educational attainment?
   1. No High School
   2. Some High School
   3. High School
   4. Some college
   5. College degree
   6. Graduate degree
9. Do you use a computer? A Mobile phone? A smart phone? A Tablet?
   1. PROBE *(interviewer to adapt probe based on the devices the participant uses)*: GO ONLINE TO USE THE INTERNET? TEXT? USE A SMARTPHONE, INCLUDING APPS? USE APPS ON YOUR TABLET?
10. What kinds of things do you do on the computer/ phone/ smart phone/ tablet? *(interviewer to adapt question based on response to #9)*
    1. PROBE (*interviewer to adapt probe based on the previous response)*: INTERNET, SCHOOL, WORK, GAMES)
    2. If not, WHY DON’T YOU USE THE INTERNET? DO YOU TEXT?
11. (If applicable) Which device do you use to go online/use the internet *most* often? (*interviewer may use the term – online vs. internet - the participant uses)*
12. A lot of people say they need a loved one to get them on the Internet, or that they can only do a few things on their own. Do you ever have help when you want to use the internet?
    1. (PROBE: FAMILY MEMBERS HELPING, CONFIDENCE WITH TECHNOLOGY)
13. Tell me about what a typical day looks like in terms of using the Internet (*interviewer may also ask about* *other devices the participant brings up).*
    1. PROBE: Do you use the internet/ go online every day? How much? If not, why not?
14. What kinds of things do you do online?
    1. PROBE: EMAIL, ONLINE/INTERNET SEARCH, SOCIAL NETWORKING, ONLINE SHOPPING, ONLINE BANKING, looking up health information
    2. PROBE: Do you ever use a credit card online? (i.e. shopping, banking, bills)
15. Do you go online/ use the Internet to look up things about your health or the health of someone in your family?
    1. PROBE: If yes, specific sites? Why those?
16. Have you ever heard of virtual reality? If so, what have you heard?
17. Have you ever used virtual reality or 3D tools?
    1. PROBE: What about gaming?
    2. PROBE: If yes, have you ever used these tools to manage your pain?

**Step 4. Interview questions to ask before using the headset**

1. First, we will ask you about your pain. This is not to formally track or analyze your pain, but rather to provide additional context to the usability test. Please rate your pain by indicating the number that best describes your pain on average in the last 24 hours, with 0 meaning ‘No pain’ and ‘10’ meaning ‘Pain as bad as you can imagine.’^1^
2. What current approaches do you take to manage your pain?
   1. PROBE: prescription medication? Over the counter medication? Injections? Acupuncture? Massage? Pacing activities? TENS units? Meditation? Prayer? Pain diaries/ classes/ support groups? Mobile applications? Something else?
   2. PROBE: What is working? What is not working?
3. What challenges have you faced in pain management? What do you think would improve this?
4. Have you heard of virtual reality to manage pain? What are your thoughts about this? Have you ever used virtual reality before?
   1. PROBE: If yes, why are you interested in this? If not, why not?

**Step 5. Usability test**

Inform patient that:

- There’s no right or wrong way to answer.
- Remind patient that they are the expert and that we want their feedback
- We didn’t design the product so the patient will not offend us with their honest feedback
- We will observe them, and just want them to talk as much as they feel comfortable
- If at any time they want to take the headset off, or feel uncomfortable, they should do so or let us know they would like to stop
- We will try a 10 minute program, but you can stop before then if you want.

**Step 6. Interview questions to ask after using the headset**

1. What did you think about that experience?
   1. PROBE: Were there any parts that surprised you? Did you think it was relevant?
2. What did you think about putting the headset on?
   1. PROBE: Did you experience any side effects? What about motion sickness?
3. What did you think about what you watched and heard while wearing the headset?
   1. PROBE: Would you have preferred to watch or listen to something different? What would that be?
4. What would you change about the headset?
   1. PROBE: The way it fits? The way it is designed? What about how it feels on your head?
   2. PROBE: Would you design it differently? What would you change?
5. Would you be interested in trying it again?
   1. PROBE: At home? During clinical care? As a replacement for some pain management strategies?
   2. PROBE: What challenges might you face in using this?
6. Do you think other patients would be interested in trying this? Why or why not?
   1. PROBE: what challenges might they face in using something like this? What might they appreciate?
7. Would you recommend VR to a friend?
8. (*interviewer to ask this question 5-10 minutes after headset use):* I would like to ask you about your pain again. This is not to formally track or analyze, but to provide context to the usability test. Please rate your pain by indicating the number that best describes your pain on average in the last 24 hours, with 0 meaning ‘No pain’ and ‘10’ meaning ‘Pain as bad as you can imagine.’^1^
9. Overall, what are your impressions?

- **Neutral prompts**
- That’s interesting; could you say a bit more about that?
- What are you thinking now?
- What makes you say that?
- Could you tell me more about that?
- Why do you think that?
- What do you think about that?

REFERENCES

1. Cleeland CS, Ryan KM. Pain assessment: global use of the Brief Pain Inventory. *Ann Acad Med*. 1994;23:129-138
